# The challenging concept of eradication: A core concept guiding and frustrating public health

**DOI:** 10.17269/s41997-024-00947-w

**Published:** 2024-10-02

**Authors:** Arthur Caplan, Nathaniel Mamo

**Affiliations:** Division of Medical Ethics, Department of Population Health, NYU Grossman School of Medicine, New York, NY USA

**Keywords:** Poliomyelitis, Disease eradication, Global health, Vaccines, Communicable diseases, Poliomyélite, éradication de maladie, santé mondiale, vaccins, maladies transmissibles

## Abstract

The celebrated 1980 announcement that smallpox had been eradicated was made using the following definition of eradication: “Permanent reduction to zero of the worldwide incidence of infection caused by a specific agent as a result of deliberate efforts: intervention measures are no longer needed.” Public health around the world works with this definition of “eradication,” setting it as a goal for other infectious disease control programs. The definition is simple. Its application, however, has produced long-running and complex public health campaigns that threaten the commitment of funders, health care providers, and governments. In this paper, the authors demonstrate the disease-specific challenges of eradication through the example of the Global Polio Eradication Initiative (GPEI). While many deem eradication worth its high costs because it is the end of morbidity and mortality from a disease, it does not mean the end of disease control efforts. Public health must be prepared for the possibility of disease reoccurrence in the form of undetected natural reservoirs of disease, lab leaks from stored samples, bioterror attacks using stolen samples, and the synthetic recreation of microbes. This paper clarifies the role of reoccurrence prevention in eradication, calling for its addition in the definition of eradication.

## Résumé

L’annonce célèbre de 1980 que la variole a été éradiquée utilisait la définition d’éradication suivante : « Réduction permanente à zero de l’incidence mondiale d’infection causée par un agent spécifique en conséquence d’efforts délibérés : mesures d’intervention ne sont plus nécessaires. » La santé publique globale emploie cette définition de « éradication », la fixant comme objectif pour d’autres programmes de contrôle des maladies transmissibles. La définition est simple. Pourtant, son application a produit des campagnes de santé publique complèxes et de longues durées, qui menacent l’engagement des bailleurs de fonds, les prestataires des soins de santé, et les gouvernements. Dans cet article savant, les auteurs démontrent les obstacles d’éradication spécifiques à une maladie transmissible à travers l’exemple de la Global Polio Eradication Initiative (GPEI). Tandis que l’éradication pourrait peut-être mériter les côuts élevés associés puisqu’elle signifie la fin de la morbidité et la mortalité d’une infection, ceci ne représente pas la fin des efforts de contrôle des maladies. La santé publique doit se préparer pour la possibilité de la réapparition d’une maladie infectieuse en forme de reservoir de maladie naturel non-détecté, fuites de laboratoires des échantillons conservés, attaques bioterroristes utilisant des échantillons volés, et la récréation synthétique des microbes. Cet article savant clarifie le rôle de la prévention de réocurrence dans l’éradication, et demande que ce role soit ajouté à la definition d’éradication.

Theoretical discussions debated what the definition of “disease eradication” should be after its failed application for malaria, yaws, and yellow fever (Dowdle, 1998, p. 22). The 10-year WHO Smallpox Eradication Programme (SEP) defined eradication as the reduction of the worldwide burden of smallpox to zero cases, which it achieved in 1980 (Henderson, 2009). The world subsequently rejoiced and dropped smallpox from the public health agenda entirely. It is the only available example of successful eradication of a human disease, and unified consensus around a definition.

Walter R. Dowdle, a former Centers for Disease Control (CDC) Director, defined eradication as follows: “Permanent reduction to zero of the worldwide incidence of infection caused by a specific agent as a result of deliberate efforts: intervention measures are no longer needed” (Dowdle, 1998, p. 23). This definition is how the goal for smallpox eradication was formulated, and how the goal has been defined in public health for succeeding eradication programs (CDC, 1993). All eradication programs since smallpox have used this definition, but none have succeeded in meeting it. This paper will explore the challenges of applying this definition through the example of polio, and clarify that eradication is not the end of disease control efforts, to better assess its value to public health.

Smallpox eradication was the direct impetus for the WHO creating the Global Polio Eradication Initiative (GPEI) in 1988. Its original goal was polio eradication by 2000, a goal that still has not been met. Its delay is because of biological challenges and changing social conditions that make meeting the definition of eradication extremely difficult. The biology of polio is very different from that of smallpox, which, despite a huge reduction in cases, continues to spread.

The vast majority of polio cases, around 70%, are subclinical with no symptoms (Walter & Malani, 2022). Smallpox, on the other hand, presented itself every time with a distinctive rash (Orenstein et al., 2022). Polio surveillance systems cannot directly detect cases unless they are paralytic, which involves fewer than 1% of cases (GPEI, 2023; Walter & Malani, 2022). Because of polio’s high incidence of subclinical cases, health system observers have a limited ability to know the transmission levels in a community. Even in the most well-run health systems, cases can be missed, convincing observers into thinking polio is gone. In nations like Somalia or Yemen, where surveillance is complicated by political instability, and in refugee camps lacking basic healthcare, there is little chance of accurate detection. Achieving eradication relies on questionable surveillance in sometimes difficult conditions that allow for plenty of places a polio case can go undetected, until a larger, possibly even epidemic, reoccurrence.

Yet another difference from smallpox which has made polio eradication very difficult is that the most common affordable polio vaccine introduced an unexpected biological challenge. The oral polio vaccine (OPV) is an overwhelmingly safe and effective vaccine (Orenstein et al., 2022). Its only significant adverse event, however, is that it (very rarely) transmits polio. OPV can cause vaccine-derived poliovirus that is no different from the wild poliovirus (Burns et al., 2014). The best weapon against polio, ironically, was eventually found to cause polio (Abraham, 2018).

GPEI has recently sought technological solutions to these biological challenges to move on with the eradication goal. It has experimented with a cheaper and faster surveillance system, and has widely implemented a novel oral polio vaccine (nOPV) that has a reduced risk of vaccine-derived poliovirus (GPEI, 2021, p. 25; Shaw et al., 2020). The technological responses now make eradication more feasible.

Other realities besides biology confound polio eradication. A very high percentage of the world population needs to be vaccinated against polio to achieve eradication. Many of the unvaccinated are in very remote areas of the world, isolated by or fleeing civil conflict and served by flimsy health systems. Even access does not in today’s world mean uptake. Increasingly, since the COVID-19 pandemic, worldwide trust in vaccines has declined, partly because of the spread of vaccine misinformation (Hotez, 2023). Polio might never be eradicated just because not enough people want the vaccine. Public health’s approach to polio, unlike with smallpox where support was nearly universal and occasional hesitancy could be met with compulsory vaccination, faces a new moral challenge (Greenough, 1995). Now, widespread vaccine hesitancy exists in an ethical climate in which many detest compulsory vaccination, limiting public health’s options. Still, many believe aiming for eradication is doable and worthwhile (GPEI, 2021, p. xii).

Some have favoured eradication because they believe it means that disease control efforts could be suspended and the money saved redistributed. The anticipated savings are sometimes used to justify the decades-long costs of eradication campaigns. But, eradication does not mean, as many have thought, abandoning all efforts at control once zero cases are detected. It is important to understand that the concept of eradication is a process, not an event, with continuing costs.

Eradication is not, as the definition is sometimes interpreted to mean, the moment when no cases are observed (Dowdle, 1998, p. 23). Dowdle’s condition that “intervention measures are no longer needed” has been assumed to include all activities. Because we do not get vaccinated against smallpox and there have been no new cases, many will make this assumption. But once there are zero cases, new measures need to be established to meet the threat of reoccurrence in humans. Eradication is not “extinction”, a state where the disease-causing pathogen no longer exists (Dowdle, 1998, p. 23). At eradication, the pathogen exists separately from the manifestation of disease, creating the threat of reoccurrence. Populations without ongoing surveillance and a strategy for detection and rapid intervention become vulnerable to reoccurrence in the form of undetected natural reservoirs of disease, lab leaks from stored samples, bioterror attacks using stolen samples, and the synthetic recreation of microbes.

The last reported case of wild smallpox was in Somalia in 1977 (Henderson, 2009). Of any smallpox, the last case was in 1978 in Birmingham, United Kingdom, when a hospital worker was infected and died from smallpox samples kept at a laboratory (Williams, 2020). The worker had not been recently vaccinated because the UK ceased smallpox vaccinations after it had eliminated the disease (Williams, 2020). By then, the world had not seen a smallpox case in over a year. But despite the end of prevention measures, there was still a real risk of reoccurrence, which the lab accident proved. The same could happen today, as samples of *variola virus* are still stored in a few laboratories. Poliovirus samples will remain after “eradication”.

Preventing reoccurrence calls for sustained disease prevention measures post apparent eradication. A response strategy must be designed and ready to be deployed in the case of reoccurrence. Maintaining eradication permanently must be added in the definition of eradication. Because what is permanent yesterday may not be today or tomorrow.

A growing number of disease experts recognize and plan for this. In 2018, GPEI published its provisional plan for the world after polio eradication is certified (WHO, 2018). Significant planning has been done to manage the risks of reoccurrence and to set up robust systems, nationally and globally, to respond to an episode. At the certification of eradication, the GPEI will transition activities to the “future owners” of polio eradication (WHO, 2018, p. 10). National governments will take a much larger role for the maintenance of eradication. The next stage relies on understanding that eradication requires indefinite activities.

When smallpox reoccurred in Birmingham, the city shut down and ring vaccination followed (Williams, 2020). Only two people got sick and one died in a city of over a million (Williams, 2020). Strong public health systems, vigilance, and response prevented the world from being victim again to an “eradicated” horrific disease. The eradication of other diseases will require the same.

The definition of eradication says nothing about the chances of eradicating and maintaining eradication for any one disease. The polio campaign has shown some of the tough issues that eradication efforts can encounter. Smallpox eradication was done with a very different disease, during a time when community interests trumped an individual’s and when the capacity for bioterror was less sophisticated. Polio and other diseases have complicated the concept of eradication.

Infectious disease eradication is the greatest gift public health has to offer. However, the effort of global public health to attain this goal is wasted if it is misunderstood. Eradication is worthwhile because the benefits of eradication far outweigh its costs, but eradication must be clearly defined today for non-experts, funders, and the public as to what permanency requires: engaging with shifting ethical norms about consent to vaccination, the new threats of bioterror, the costs of maintaining surveillance vigilance, and the capacity to respond rapidly to any reoccurrence.

## Data Availability

Not applicable.
